# Delayed age at transfer of adoptees to adoptive parents is associated with increased mortality irrespective of social class of the adoptive parents: a cohort study

**DOI:** 10.1186/s12889-018-5338-4

**Published:** 2018-04-24

**Authors:** Liselotte Petersen, Per Kragh Andersen, Thorkild I. A. Sørensen, Erik Lykke Mortensen

**Affiliations:** 1 National Centre for Register-based Research, Aarhus University, Fuglesangs Allé 26, DK-8210 Aarhus V, Denmark; 2 Department of Public Health, Faculty of Health and Medical Sciences, University of Copenhagen, Øster Farimagsgade 5B, DK-1014 Copenhagen K, Denmark; 3 Novo Nordisk Foundation Center for Basic Metabolic Research, Faculty of Health and Medical Sciences, University of Copenhagen, Copenhagen, Denmark; 4 Center for Healthy Aging, Faculty of Health and Medical Sciences, University of Copenhagen, Øster Farimagsgade 5B, DK-1014 Copenhagen K, Denmark

**Keywords:** Adoption, Age of transfer, Adoptive environment, Mortality, Suicide

## Abstract

**Background:**

Adverse early life experience and development may have long-term health consequences, but later environmental conditions may perhaps protect against the effects of such early life adversities. The aim was to investigate whether cause-specific and overall mortality rates among adoptees are associated with the age at which they were transferred to the adoptive family and whether the social class of the adoptive family modifies this association.

**Methods:**

A cohort of 10,592 non-familial adoptions (biologically unrelated adoptee and adoptive parents) of Danish-born children formally granted in 1924–47 and with follow-up of total and cause-specific mortality through ages up to 85 years. The rates of death after the age of 16 from all causes combined, all natural causes, all external causes, and suicide were compared according to the age at which adoptees were transferred to their adoptive family by estimating hazard ratios in Cox regression models.

**Results:**

Death rates from all causes were significantly higher in adoptees transferred between age 1 month and 4 years compared to those transferred immediately after birth with the hazard ratio peaking at 1.19 (95% confidence limit: 1.08 to 1.32) for adoptees transferred between 6 and 11 months. This result was primarily driven by a similar pattern for natural causes of death. For death from external causes and for suicide the hazard ratios were increasing with increasing age at transfer, and tests for trend were statistically significant. The social class of the adoptive family did not significantly modify these associations.

**Conclusions:**

Transfer to an adoptive family later than at the time of birth may have adverse long-term consequences affecting overall and cause-specific mortality. These effects were not modified by the environment provided by the adoptive family as indicated by the social class of these families.

**Electronic supplementary material:**

The online version of this article (10.1186/s12889-018-5338-4) contains supplementary material, which is available to authorized users.

## Background

Adverse early life experience and development may have long-term health consequences [1], but later environmental conditions may protect against these effects. Studies of children adopted to biologically unrelated families may contribute to elucidate these effects by assessment of the associations with timing of transfer to the adoptive family, of change in environment, and the importance of the adoptive environment. While most follow-up studies of adopted children have focused on behavioral, cognitive and emotional development [2–4], a number of Danish studies have focused on mortality in adoptees [5–7]. Compared with the general population, adoption was found to be associated with increased rates of both all-cause mortality and of specific causes of death, such as infections, vascular disease and cancer as well as alcohol-related deaths and suicide [5]. The increased mortality among the adoptees in general may reflect early adversities associated with genetic and/or environmental family factors, factors related to institutional rearing, and factors associated with transfer to a new family and growing up with new primary caretakers.

Miller et al. discussed associations between exposure to major psychological stressors in early life and elevated rates of morbidity and mortality from chronic diseases of aging [1]. They suggested mechanisms from early stress through altered patterns of endocrine and autonomic discharge resulting in inflammation, which drives forward pathogenic mechanisms that ultimately foster chronic disease. If early adversities are important determinants of health in adoptees, older age at the time of transfer to the adoptive family should be associated with increased mortality. Later age of transfer has in fact been found to be associated with less optimal physical [8] and mental development [3, 4, 9], but later age of transfer could also be associated with disruption of established relationships with caretakers and difficulties in forming attachment relationships with the new primary caretakers [10–13]. While most adopted children seem to be able to establish new relationships [14–16], change in family and primary caregiver should still be considered a major childhood stressor, which may not only influence the child’s ability to establish secure attachment relationship, but also the child’s long-term cognitive, personality and social development. This may explain the increased risk of behavior problems often observed in adopted children [2, 3, 9], which potentially also have long-term consequences for lifestyle, morbidity and later mortality [17, 18].

Socioeconomic circumstances during up-bringing influence cause-specific and overall mortality in adulthood [19]. It is therefore likely that higher social class of adoptive parents could compensate for the detrimental influence of later age of transfer on morbidity and mortality in adoptees. Thus, high social class parents may provide an environment that could counteract the effects of early adversities, and the stress associated with change in primary caretakers. High adoptive social class may therefore be associated with fewer behavior problems in childhood and adolescence, more optimal physical and mental development as well as more healthy lifestyles followed by lower morbidity and mortality. However, a previous study showed that the social class of the adoptive father was unrelated to the adoptee’s rate of dying from natural causes, whereas the incidence of suicide was increased among those with a high social class adoptive father [20]. These associations may well depend on the age of transfer to the adoptive parents and the circumstances leading to the adoption.

The Danish Adoption Register includes information on adoptions of Danish-born children granted in 1924–47 [21]. Adoptees were transferred to a biologically unrelated adoptive family and the adoption register therefore provides an opportunity to explore long-term consequences of age of transfer into a new family. The baseline data collected from administrative archives are somewhat limited, but in combination with the Danish cause of death registration they provide unique possibilities to investigate mortality patterns many years later. The aim of the present study was to assess possible differences in cause-specific and overall mortality rates throughout adult life among adoptees according to the age at which they were transferred to their adoptive family. A secondary aim was to assess if the social class of the adoptive family modifies the effects of age at transfer to the adoptive family. We investigated rates of death from all causes combined, all natural causes, all external causes, and suicide by following the adoptees up to age 85 years.

## Methods

**Fig. 1:**
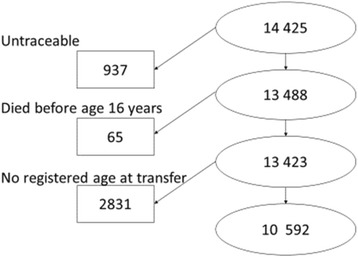
Flow chart illustrating the study population

The study was based on the Danish Adoption Register, which contains records on all 14,425 non-familial adoptions formally granted in Denmark during the period 1924 through 1947 [21]. Among the adoptees, 937 were not traceable, and 65 died before reaching 16 years of age (see flow chart in Fig. 1). The official death certificates hold information on cause of death as obtained from the Danish Register on Causes of Death. The adoptees were followed from age 16 years and until death, emigration, or until censoring at December 31, 2009, whichever came first, corresponding to follow-up to maximum ages 62 through 85 years. Among the adoptees, 2831 had no registered age at transfer, and these adoptees were excluded from the analyses, leaving 10,592 for analyses. Among those dying, 222 were not registered with a cause of death, and therefore they were censored at time of death in the cause-specific analysis. Table 1 presents the number of adoptees according to age at transfer, and the number and percentages of adoptees dying from all causes combined, from natural causes, from external causes, and from suicide.

**Table 1:** Number and percentage of deaths, according to age at transfer during the follow-up period 1940–2009

Age at transfer	Number of adoptees	Number of deaths and cause-specific deaths (%)			
		All causes	Natural	External	Suicides
At birth	2701	856 (31.7%)	706 (26.1%)	91 (3.4%)	39 (1.4%)
1–5 months	3357	1183 (35.2%)	992 (29.6%)	107 (3.2%)	50 (1.5%)
6–11 months	1732	729 (41.6%)	614 (35.4%)	66 (3.8%)	32 (1.9%)
12–23 months	1474	656 (44.5%)	555 (37.7%)	60 (4.1%)	29 (2.0%)
2–3 years	1003	498 (49.7%)	424 (42.3%)	45 (4.5%)	23 (2.3%)
4–7 years	325	179 (55.1%)	141 (43.4%)	25 (7.7%)	13 (4.0%)
No registered age of transfer	2831	1145 (40.5%)	975 (34.4%)	88 (3.1%)	45 (1.6%)
In total	13,423	5246 (39.1%)	4407 (32.8%)	482 (3.6%)	231 (1.7%)

### Categorizing age at transfer

To avoid assuming linearity we categorized age at transfer, but due to the fewer adoptees transferred after the age of two years, the categories were broader at later ages than early ages. The available studies of mortality in adoptees do not suggest specific transfer age categories, but the more detailed categorization of the first year of life used in the present study is in line with attachment theory and with a critical factor being whether the child is sufficiently old to become conscious of the transfer and the associated change in social environment.

### Measure of social class

In the present study the social class of the adoptive family was based on occupation of the fathers, and it was available from the adoption records for 96% of the adoptive fathers. In the first half of the previous century most women were housewives and it was common to define family social status by the father’s education and occupation [22].The occupations were coded as derived from a Danish study of prestige ratings of occupational titles [22]. The scale ranges from low positions (unskilled manual workers) to high positions (advanced professional). Details of the scale have been reported previously [23].

### Statistical methods

Mortality rate ratios were calculated in a Cox model with age as the underlying time variable using Stata version 12.0 [StataCorp, College Station, TX]. Calendar time was categorized in 10 year groups and adjusted for as a time-varying covariate, in order to account for changes in rate of death during the calendar period covered. Sex was adjusted for using separate underlying hazard functions in men and women, to reflect that men and women have different mortality rates.

In the main analyses, adoptees were divided into six groups according to their age at the time of transfer to the adoptive parents: 0 months, 1–5 months, 6–11 months, 12–23 months, 2–3 years, and 4–7 years. The social class of the adoptive family was considered a factor potentially modifying the effects of age at transfer and was categorized into high and low social class. High social class included advanced professional, academic and sub-academic professions and self-employed, while low social class included subordinate clerk, skilled worker, semi-skilled worker, and unskilled worker. In the analyses including the potential modifying effect of paternal social class, age at transfer was reduced to four categories: 0 months, 1–5 months, 6–23 months, and 2–7 years.

Interaction terms were added to test if gender modified the association between age at transfer and the mortality rates. The proportional hazards assumption was tested in the main analyses by Schoenfeld’s residuals and was not rejected. Linearity was tested using cubic splines, and if not rejected, tests for trend were performed assuming linearity of the age at transfer on the log hazard scale. In additional analyses age at transfer was used as a continuous variable, and cubic splines were applied in the Cox regression. As a supplementary analyses we used age at transfer to predict mortality among birth parents.

## Results

Mortality rates among adoptees were compared according to the age at which the adoptee was transferred to the adoptive family, using those who were transferred immediately after birth as the reference.

Death rates from all causes were significantly higher in adoptees transferred between age 1 month and 4 years compared to those transferred immediately after birth with the relative rate peaking at 1.19 (95% confidence limit: 1.08 to 1.32) for adoptees transferred between 6 and 11 months (Table 2). This association was primarily driven by a similar pattern for natural causes of death, where death rates from natural causes were significantly higher in adoptees transferred between age 1 month and 2 years compared to those transferred immediately after birth. For death from external causes and for suicide the mortality rates were higher at later age at transfer, and tests for trend were statistically significant (Table 2).

**Table 2:** Relative mortality rates according to age at transfer

Age at transfer	All causes	Natural causes	External causes	Suicides
At birth	1 (reference)	1 (reference)	1 (reference)	1 (reference)
1–5 months	1.09 (1.00–1.19)	1.12 (1.02–1.23)	0.93 (0.70–1.23)	1.03 (0.68–1.57)
6–11 months	1.19 (1.08–1.32)	1.19 (1.06–1.32)	1.05 (0.76–1.44)	1.23 (0.77–1.97)
12–23 months	1.14 (1.03–1.26)	1.11 (1.00–1.25)	1.07 (0.77–1.49)	1.26 (0.77–2.05)
2–3 years	1.12 (1.00–1.26)	1.02 (0.90–1.16)	1.09 (0.76–1.57)	1.44 (0.85–2.44)
4–7 years	1.05 (0.89–1.24)	0.83 (0.69–1.00)	1.88 (1.19–2.96)	2.63 (1.38–5.01)
Overall effect	0.02	0.001	0.08	0.06
Test for trend^a^	–	–	0.006	0.003

Deaths occurred from age 16 between 1940 and 2009 among 10,592 Danish adoptees born 1924 to 1947. Analyses were adjusted for sex, age and calendar time

^a^Linearity was tested using cubic spline analysis, if not rejected test for trend was performed

We tested whether the effect of age at transfer differed in men and women and found that this was not the case for any of the causes of death (*p* > 0.16).

The number of adoptees and number and percentage of deaths from all causes of death combined, natural causes, external causes, and suicide are presented in Table 3 by age at transfer and social class of the adoptive families. Mortality rate ratios according to age at transfer are presented separately for those brought up in low and high social class adoptive families (Table 4). A test of different effects of age at transfer on death rates in those brought up in high adoptive social class and those brought up in low adoptive class did not reach statistical significance (*p* > 0.21). In adoptees who were adopted to low social class families, transfer interval 2–7 years was associated with a significantly higher rate of suicide than among those transferred at birth. This was not the case for those adopted to high social class families, but for those transferred at birth, high social class was associated with a non-significantly higher rate of suicide than among those transferred to low social class families.

**Table 3:** Number and percentage of deaths, according to age at transfer and social class of adoptive father

Social class during childhood	Age at transfer	Number of adoptees	Number of deaths and cause-specific deaths (%)			
			All causes	Natural	External	Suicides
Low social class	At birth	1772	586 (33.1%)	491 (27.7%)	59 (3.3%)	21 (1.2%)
	1–5 months	2136	795 (37.2%)	662 (31.0%)	67 (3.1%)	27 (1.3%)
	6–23 months	1920	829 (43.2%)	715 (37.2%)	67 (3.5%)	30 (1.6%)
	2–7 years	730	368 (50.4%)	301 (41.2%)	44 (6.0%)	21 (2.9%)
	No transfer age	1512	608 (40.2%)	524 (34.7%)	41 (2.7%)	18 (1.2%)
High social class	At birth	867	240 (27.7%)	193 (22.3%)	28 (3.2%)	16 (1.8%)
	1–5 months	1144	351 (30.7%)	293 (25.6%)	40 (3.5%)	23 (2.0%)
	6–23 months	1153	484 (42.0%)	396 (34.3%)	49 (4.2%)	26 (2.3%)
	2–7 years	503	251 (49.9%)	215 (42.7%)	20 (4.0%)	12 (2.4%)
	No transfer age	1191	486 (40.8%)	412 (34.6%)	40 (3.4%)	21 (1.8%)
Missing		495	248 (50.1%)	205 (41.4%)	27 (5.5%)	16 (3.2%)
	In total	13,423	5246 (39.1%)	4407 (32.8%)	482 (3.6%)	231 (1.7%)

**Table 4:** Relative mortality rate, according to their age at transfer separately for high and low social class of their adoptive father

Social class during childhood	Age at transfer	All causes	Natural causes	External causes	Suicides
Low social class	At birth	1 (reference)	1 (reference)	1 (reference)	1 (reference)
	1–5 months	1.09 (0.98–1.21)	1.09 (0.97–1.22)	0.93 (0.65–1.32)	1·07 (0·61–1·90)
	6–23 months	1.11 (1.00–1.23)	1.11 (0.98–1.24)	0.94 (0.66–1.33)	1·21 (0·69–2·13)
	2–7 years	1.06 (0.92–1.21)	0.91 (0.78–1.05)	1.50 (1.01–2.23)	2·15 (1·16–3·97)
	Overall effect	0.27	0.02	0.06	0·06
	Test for trend^a^	–	–	0.005	0·02
High social class	At birth	1 (reference)	1 (reference)	1 (reference)	1 (reference)
	1–5 months	1.11 (0.94–1.31)	1.21 (1.01–1.45)	1.07 (0.66–1.74)	1.08 (0.57–2.05)
	6–23 months	1.27 (1.09–1.48)	1.27 (1.07–1.51)	1.19 (0.74–1.90)	1.14 (0.61–2.14)
	2–7 years	1.20 (1.00–1.43)	1.13 (0.93–1.38)	0.97 (0.54–1.74)	1.17 (0.55–2.50)
	Overall effect	0.02	0.05	0.84	0.97
	Test for trend^a^	–	–	0.26	0.05
High versus low social class^b^	Transfer at birth	0.91 (0.78–1.06)	0.88 (0.74–1.04)	1.00 (0.64–1.57)	1.62 (0.85–3.11)
Test for modifying effect		0.36	0.33	0.22	0.53

Follow up from during 1940 to 2009 of 10,218 Danish adoptees born 1924 to 1947. Analyses were adjusted for sex, age and calendar time

^a^Linearity was tested using cubic spline analysis, if accepted test for trend was performed

^b^The presented estimates refer to transfer at birth. For other ages of transfer, the hazard ratio for high versus low social class must be multiplied by the estimated hazard ratio at each age at transfer. Thus, for suicide the ratio for high versus low social class for 6–23 months is 1.62 x (1.14/1.21) = 1.53

To assess confounding due to increased relative mortality in birth parents, we made a supplementary analysis of the association between age of transfer and mortality in the biological parents based on available subsamples of biological mothers (*n* = 1365) and biological fathers (*n* = 1194) [6]. Overall, this association was significant for biological mothers, but not for biological fathers; in particular, transfer at age 4–7 years was associated with higher mortality in the biological mother and perhaps the biological father also (Additional file 1: Table S1).

## Discussion

An association of age at transfer with overall and cause-specific mortality rates was observed. For all causes combined the critical period of transfer seemed to be between 1 month and 4 years of age at transfer, while for natural causes it was 1 month to 2 years of age. For death from all external causes and for suicide the mortality rates were increasing with increasing age at transfer. The results gave no indication that the effects of age of transfer differed between those adopted to high and low social class families, although high social class was associated with a non-significantly higher rate of suicide.

The pattern found for natural causes suggested a difference between transfer at birth and later transfer. The lack of a clear trend of increasing mortality with increasing age of transfer suggests no cumulative effects of adversities during the first 7 years of life. For the rate of suicide and all external causes the trend is significant, but a pattern of increasing rate with increasing age is most clear for suicide. This may be related to the behavior and emotional problems that have been observed in many studies of adopted children and adolescents. Thus, while some studies of international adoptees have found late age of transfer to predict behavioral and emotional problems [9, 24, 25], these studies also suggest that later age of transfer is associated with greater likelihood of exposure to psychosocial adversities [26] or longer institutional rearing [27, 28]. These findings were only partially corroborated in a US study of 1948 internationally adopted children where adoption at age 2 years or later predicted both externalizing and internalizing behavior problems independent of institutional rearing [25]. A Finnish study of 1265 international adoptees found no linear effect of time of arrival to Finland [29]. A possible effect of early adversities may change with age at assessment, Sonuga-Barke et al. found a catch-up on cognitive impairment, but increasing emotional problems before young adulthood [9]. Other sources of inconsistency between studies could be limited power. Thus, some studies are very small and included only 36, 108 and 124 adoptees [10, 12, 16] and this restricts the opportunity to model the effect of age at transfer appropriately.

Our study of domestic adoptees born in the first half of the twentieth century represents circumstances that are very different from those of international adoptees at present. In our study, neither the reasons for adoption, nor information on institutional rearing before transfer to the adoptive family were available but a number of different factors may predict behavior problems and the increase in mortality from natural causes associated with transfer at 1 month or later. Familial environment such as parental attachment patterns has been found to affect attachment in adoptees [30] and could be linked to mortality but in our study detailed information on family environment in the adoptive family was not available, and SES was used as an indicator of the family environment. A remarkable finding is that the analyses did not support the hypothesis that adoption to high social class families compensates for the increased mortality rate from natural causes associated with transfer at 1 month or later; if anything, the tendency was the opposite. Similarly, there was no evidence for death from external causes that social conditions may compensate for late age at transfer, and in fact the rate of suicide was higher for those brought up in high social class compared with those brought up in low social class adoptive families, a finding previously observed by Osler et al. in the same study population [20]. Thus, this relatively large study provides little evidence that the social class of the adoptive family influences mortality from natural causes or modifies the effect of age of transfer.

The important contrast for age at transfer seems to be between transfer at birth and later in childhood, and thus there is little evidence of cumulative effects of adverse psychosocial circumstances before adoption on the rate of death from natural causes. An alternative interpretation is that attachment is affected differently in different developmental periods. Thus, age at transfer is likely to correlate with adversities and prolonged sojourn in institutions, but with the information available the different explanations for our findings cannot be disentangled. The period of vulnerability to change in family differed between natural and external causes of death, and this suggests that the mechanisms leading from timing of transfer into the adoptive family to increased rate of death may be different for natural and external causes of death, in particular suicide. For natural causes of death it is possible that the difference between adoption at birth and later reflects differences in health related characteristics between the biological parents of children transferred early and late. Thus, late transfer was often associated with prolonged sojourn in institutions related to maternal inability to take care of the child [31, 32], and the supplementary analysis suggested an association between late transfer and early death of the biological mother. The late transfer is likely due to illness in birth mother, as indicated by their increased mortality according to late transfer. The risk of suicide may be related to psychosocial adversities before adoption as well as an association between later age of transfer and difficulties related to the change of environment and integration in the new family. In an observational cohort study with limited information at baseline, it will of course be difficult to make counterfactual inferences about what would have happened to the children if they had not been adopted away and transferred to the adoptive family at a certain age. The conclusions from the study is based only on the comparisons of adoptees with different ages at transfer to the adoptive families.

## Conclusions

We find that transfer to an adoptive family at a later time than at birth may have long-term consequences affecting mortality. Being transferred later than birth was associated with a higher rate of death from natural causes, whereas the later the age at transfer, the higher the rate of suicide and other externally-caused death. These effects were not modified by the environment provided by the adoptive family as assessed by the social class of these families.

## Additional file

Additional file 1: **Table S1.** Associations between age of transfer and mortality in the biological parents. (DOCX 37 kb)
