# Supplementary material for: Delayed age at transfer of adoptees to adoptive parents is associated with increased mortality irrespective of social class of the adoptive parents: a cohort study
Source: BMC Public Health. 2018 Apr 24;18:435. doi: 10.1186/s12889-018-5338-4 (PMC5914045; doi:10.1186/s12889-018-5338-4)
Supplement: Supplementary file 1 — Table S1. Associations between age of transfer and mortality in the biological parents. (DOCX 37 kb) [file 12889_2018_5338_MOESM1_ESM.docx]

**Additional file 1: Table S1**

Associations between age of transfer and mortality in the biological parents.

| Age at transfer | All causes, BM  N=1365, events=986 | All causes, BF  N=1194  events=1032 |
| --- | --- | --- |
| At birth | 1 (reference) | 1 (reference) |
| 1-5 months | 1.05 (0.89 – 1.25) | 1.08 (0.92 – 1.28) |
| 6-11 months | 0.93 (0.76 – 1.15) | 1.16 (0.95 – 1.41) |
| 12-23 months | 1.30 (1.06 – 1.60) | 1.15 (0.94 – 1.42) |
| 2-3 years | 1.07 (0.84 – 1.36) | 1.05 (0.82 – 1.35) |
| 4-7 years | 1.50 (1.07 – 2.10) | 1.21 (0.87 – 1.70) |
| Overall effect | 0.01 | 0.65 |
| Test for trend* | - | - |

Mothers are followed from birth of the adoptee, and the analysis is adjusted for age, year of birth in 7 groups. Fathers are followed from conception of the adoptee, and the analysis is adjusted for age and year of birth in 7 groups.
